# Improvement in symptom-related disruptions is associated with fewer days of short-acting beta-agonist use in asthma

**DOI:** 10.1038/s41533-022-00299-3

**Published:** 2022-09-02

**Authors:** Leanne Kaye, Vy Vuong, Meredith A. Barrett, Elroy Boers, Theresa Guilbert

**Affiliations:** 1 ResMed Science Center, San Diego, CA USA; 2 ResMed Science Center, Halifax, NS Canada; 3 Department of Pediatrics, University of Cincinnati and Pulmonary Division, Cincinnati Children’s Hospital Medical Center, Cincinnati, OH USA

**Keywords:** Asthma, Epidemiology

## Abstract

Significant indirect healthcare costs are related to uncontrolled asthma, including productivity loss. Days with short-acting beta-agonist (SABA) use is associated with symptom-related disruptions at work, home, and school. Digital self-management platforms may support fewer days with SABA medication use and may reduce symptom-related disruptions.

## Introduction

Poorly controlled asthma, representing ~60% of children and adults with asthma in the United States^1^, can result in significant healthcare costs and lost productivity at school, work, and home, of which the latter is estimated to cost $3B annually^2^. A similar loss in productivity is also observed globally^3^. Digital health solutions have shown promising results in promoting improved clinical outcomes and reducing healthcare resource utilization in asthma^4,5^, but few studies have explored its potential to assess productivity with passively-collected electronic data.

Electronically-recorded short-acting beta-agonist (SABA) medication use may serve as an important objective determinant of asthma control, as shown in Anderson et al.^6^ who found that electronically-recorded SABA usage was correlated with self-reported SABA use as captured by question 4 of the Asthma Control Test (ACT). Further, electronic tools like electronic medication monitors (EMMs) could reduce recall bias associated with self-reported symptom surveys like the ACT or the Asthma Control Questionnaire. We hypothesized that electronically-recorded days without use (“SABA-free days”, SFD) may also act as an important objective proxy for productivity. As such, this study aimed to understand the relationship between electronically-captured SFD and self-reported productivity, representing symptom-related disruptions at work, school, and home from question 1 of the ACT, and how these outcomes change over time when enrolled in a digital self-management platform.

## Results

### The relationship between ACT Q1 and SABA-free days

**Fig. 1:**
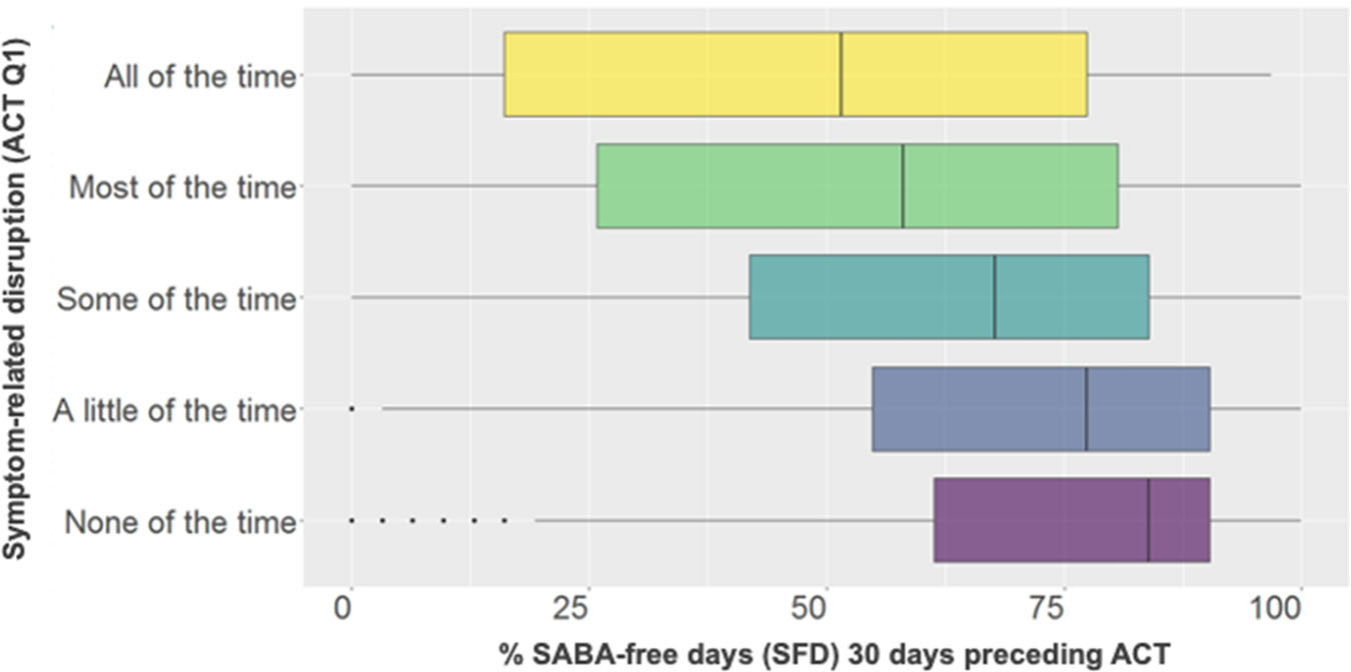
Self-reported productivity was significantly associated with SABA-free days. Box plots of self-reported productivity and SABA-free days. Box-plot elements include: center line is the median value; the box lower and upper limits correspond to the first and third quartiles; the upper whisker extends from the upper limit to the largest value no further than 1.5 × interquartile range (IQR). The lower whisker extends from the lower limit to the smallest value, at most 1.5 × IQR of the hinge. Data beyond the end of the whiskers are outliers and shown individually.

In the first analysis assessing the relationship between ACT Q1 and SFD, 3,322 adolescents and adults were included (73.8% female, mean (SD) age: 40.7 (13.8) years). Self-reported symptom-related disruption was significantly associated with SFD, with higher disruption observed when fewer SFD were noted (Fig. 1). The lowest symptom-related disruption response (“none of the time” in the ACT) was reported by 1,102 patients for a median (IQR) of 83.9% (61.3, 90.3%) of SFD in the 30 preceding days. 169 patients reported the highest symptom-related disruption response (“all of the time” in the ACT) with a median (IQR) of 51.6% (16.1, 77.4%) of SFD.

All of the pairwise comparisons from the Kruskal–Wallis’s Dunn’s post hoc test were significant (*p* < 0.001), with the exception of “all of the time” and “most of the time” (*p* = 0.18) (Table 1). Moreover, the association between ACT Q1 and SFD is further confirmed by the adjusted ordinal logistic regression analysis. Specifically, we observed a rise of 2.0% in the odds of increased productivity (i.e., fewer symptom-related disruptions) for every additional SFD (OR 1.02 (95% CI: 1.01, 1.03; *p* < 0.001), keeping all other factors constant.

**Table 1:** Pairwise comparisons to determine if the percent of SFDs in the 30 days preceding an ACT was statistically different between the five response options of ACT Q1.

Pairwise comparison	*z*-score	*p* value
A little of the time - All of the time	8.2	<0.001
A little of the time - Most of the time	10.3	<0.001
All of the time - Most of the time	−1.3	0.18
A little of the time - None of the time	−3.6	<0.001
All of the time - None of the time	−9.8	<0.001
Most of the time - None of the time	−12.5	<0.001
A little of the time - Some of the time	7.5	<0.001
All of the time - Some of the time	−4.5	<0.001
Most of the time - Some of the time	−4.6	<0.001
None of the time - Some of the time	10.5	<0.001

### Changes in ACT, ACT Q1, and SABA-free days over time

In the second analysis assessing changes in ACT, ACT Q1, and SFD over 90 days, 1595 adolescents and adults were included (75.1% female, mean (SD) age: 41.5 (13.8) years, 86.6% uncontrolled asthma at enrollment (ACT≤19)). From enrollment to follow-up, the percentage of SFD increased by 10% (median IQR: 70% (43, 87%) vs. 80% (57, 90%); *p* < 0.001). Similarly, symptom-related disruptions improved across all responses to ACT Q1 (all *p* < 0.01) from enrollment to follow-up. Total ACT score also improved (median (IQR): 14 (11, 17) vs. 17 (13, 21); *p* < 0.001), with 46.3% of patients demonstrating a clinically meaningful improvement, defined as ≥3 point increase in total ACT score (Table 2).

**Table 2:** Change in self-reported productivity, SABA-free days, and ACT over 90 days.

	Baseline	Month 3 follow-up	*p* value	Test statistics	Effect size
**n**	1595	1595	-		
**% SABA-free days; median (IQR)**	70.0 (43.3, 86.7)	80.0 (56.7, 90.0)	<0.001	*W* = 307,604	*r* = 0.33
Uncontrolled (Baseline ACT ≤19), *n* = 1382	70.0 (40.0, 83.3)	76.7 (53.3, 90.0)	<0.001	*W* = 223,214	*r* = 0.35
Controlled (Baseline ACT >19), *n* = 213	83.3 (70.0, 90.0)	86.7 (73.3, 93.3)	0.013	*W* = 6796	*r* = 0.18
**Total ACT score; median (IQR)**	14 (11−17)	17 (13–21)	<0.001	*W* = 242192	*r* = 0.44
**Self-reported productivity interruption (Q1 of ACT);** ***n*** **(%)**					
All of the time	95 (6.0)	55 (3.4)	<0.001	*χ*^2^ = 14, df = 1	Cohen’s g = 0.18
Most of the time	312 (19.6)	173 (10.8)	<0.001	*χ*^2^ = 58, df = 1	Cohen’s g = 0.21
Some of the time	618 (38.7)	419 (26.3)	<0.001	*χ*^2^ = 65.2, df = 1	Cohen’s g = 0.16
A little of the time	394 (24.7)	522 (32.7)	<0.001	*χ*^2^ = 28.1, df = 1	Cohen’s g = 0.11
None of the time	176 (11.0)	426 (26.7)	<0.001	*χ*^2^ = 162.8, df = 1	Cohen’s g = 0.33

*W* is the test statistic and *r* is the effect size of the Wilcoxon signed-rank test. The effect size *r* is calculated as *Z* statistic divided by the square root of the sample size (N) (*Z*/*N*‾‾√). *N* corresponds to the total number of pairs for paired samples test.

*χ*^2^ (Chi-squared), df (degrees of freedom) is the test statistic, and Cohen’s g is the effect size of McNemar’s test. For a 2 × 2 table, where a and d are the concordant cells (the frequency of individuals who responded positively or negatively on both time points) and b and c are discordant cells (the frequency of individuals who responded differently): *P* is the greater of (b/(b + c)) or (c/ b + c)); and Cohen’s g is *P* - 0.5.

## Discussion

This study observed that electronically-captured SABA-free days were associated with symptom-related disruptions at work, school, and home. Further, the use of a digital platform was associated with a significant improvement in SFD and a reduction in monthly self-reported symptom-related disruptions in adolescents and adults with asthma.

Such insights may be important, especially when considering the indirect costs of uncontrolled asthma^7–9^. In the US, the cost of missed days of school and work for people with asthma is estimated to be $3B/year^2^. In our study, we observed that over 90 days, patients had a 10% increase in SFD (or nine SFDs (10% increase × 90 days)) and a 2% reduction in symptom-related disruptions associated with each additional SFD. As such, we calculated an 18% reduction (2% × nine more SFDs) in symptom-related disruptions, which could be crudely translated to a yearly cost savings of $540 M ($3B/year × 18%) assuming a constant rate in the reduction in disruptions.

Several studies have already demonstrated that the use of digital tools can also support asthma management, mainly by increasing adherence to treatment, identifying therapeutic failure to intervene early, or informing therapeutic adjustments^4–6,10^. The COVID-19 pandemic has further highlighted the value of remote monitoring, where connected physiologic or therapeutic monitoring can be used to support patient care when in-person clinic visits are not always feasible.^11^ While barriers to remote care adoption still exist among patients and healthcare providers, the pandemic has presented an opportunity to address these constraints and refine current digital health approaches to better support the current standard of care in asthma.

The present study demonstrates the value of electronically-captured medication use, but there are several limitations to consider. First, patients self-reported their history of asthma physician confirmation was not sought. Patients may also have used multiple inhalers, not all of which had an EMM, thus possibly preventing the assessment of complete medication usage. ACT Q1 is a self-reported measure and relies on perceived days of symptom-related disruptions and should be confirmed with additional productivity data. Further, patients included in these analyses may have been more motivated, possibly overestimating the associations observed. Future studies should explore the present findings in a larger sample size, confirm findings in more robust study designs that include a comparison group, and consider the impact of controller medication use and disease severity subgroups.

SFD may be an important objective proxy for perceived symptom-related disruptions, allowing for more regular quantification of disease impact compared to self-reported measures, which may not be completed regularly and face self-report biases. We also observed the continued clinical benefit of digital health platforms, with improvements in asthma control (ACT), productivity (ACT Q1), and SFD over the study period. Digital platforms may provide important insights into patient medication use, which can be used to enhance patient care.

## Methods

### Study design

This analysis used retrospective data collected from patients aged ≥12 years with a self-reported history of asthma who utilized a digital self-management platform (Propeller Health, Madison, WI, USA)^5^ between 2016 and 2019. Patients self-enrolled at health fairs and via social media campaigns or were recruited through clinical programs offered at their healthcare organizations. Patients did not receive monetary compensation for their participation.

### Data collection

To set up the digital platform, patients attached an EMM to their SABA inhaler to passively record the date and time of each actuation. The days in which the patient did not use their SABA medication were classified as SFD. Patients also paired their EMM with a companion smartphone app, which helped patients track medication usage and trends and provided evidence-based educational content^5^.

At enrollment and every month thereafter, patients were prompted to complete an Asthma Control Test (ACT) in the app to assess their own perception of daily functioning, symptom-related disruption, and symptom control in the four preceding weeks^12^. The first question of the ACT (ACT Q1) focuses on self-reported symptom-related disruptions, asking, “In the past 4 weeks, how much of the time did your asthma keep you from getting as much done at work, school, or at home?”. Responses range from 1 (all of the time) to 5 (none of the time).

### Ethics approval

All patients agreed to Propeller’s Terms of Use, which allows for retrospective analysis of de-identified aggregate data. The current retrospective analysis proposal was determined to be exempt by the Copernicus Institutional Review Board (PRH1-18-132).

### Retrospective analysis

The relationship between ACT Q1 and SFD was assessed cross-sectionally in patients with ≥30 days of continuous EMM data, which captured SABA use preceding a completed ACT. The Kruskal–Wallis test with Dunn’s post hoc comparison test was used to determine if the percent of SFDs in the 30 days preceding an ACT statistically differed between the five response options of ACT Q1 (all of the time, most of the time, some of the time, a little of the time, none of the time). To account for the multiple statistical tests performed simultaneously, Bonferroni correction was applied for Dunn’s post hoc comparison. We further explored the association between ACT Q1 and SFD by performing an ordinal logistic regression model adjusting for age, gender, baseline ACT, season, enrollment site, and controller inhaler use.

The change in total ACT score, ACT Q1, and SFD over time was evaluated between enrollment and month 3 (days 68–97). Patients needed to have ≥90 days of continuous EMM data, ≥1 EMM-recorded SABA usage, and 2 completed ACTs (at baseline and 3 months) to be included in the analysis. Changes over time in the total ACT and SFD were assessed using Wilcoxon signed-rank test for paired data (e.g., consisting of repeated measurements). For the changes over time in ACT Q1, the Stuart-Maxwell test checked the overall difference of all five response options to ACT Q1 at baseline and at month 3. After that, McNemar’s tests were used to test if there is a statistically significant change between baseline and month 3 in each level.

All statistical tests were two-tailed with an alpha = 0.05 threshold for statistical significance, except for Dunn’s post hoc comparison test (alpha = 0.005). All analyses were conducted in R version 4.1.1 (R Foundation for Statistical Computing).

### Reporting summary

Further information on research design is available in the Nature Research Reporting Summary linked to this article.

## Supplementary information

REPORTING SUMMARY

## Data Availability

De-identified data are available upon request with appropriate regulatory and third-party authorization.

## Code availability

Code will be made available upon request.
